# Motor flexibility to stabilize the toe position during obstacle crossing in older adults: an investigation using an uncontrolled manifold analysis

**DOI:** 10.3389/fspor.2024.1382194

**Published:** 2024-03-22

**Authors:** Yuki Suda, Kentaro Kodama, Takahito Nakamura, Juntaro Sakazaki, Takahiro Higuchi

**Affiliations:** ^1^ Department of Health Promotion Science, Tokyo Metropolitan University, Tokyo, Japan; ^2^ Japan Society for the Promotion of Science, Tokyo, Japan; ^3^ University Education Center, Tokyo Metropolitan University, Tokyo, Japan; ^4^ Department of Physical Therapy, School of Health and Social Services, Saitama Prefectural University, Saitama, Japan

**Keywords:** uncontrolled manifold analysis, kinematic redundancy, adaptive locomotor adjustment, conservative strategy, toe clearance, obstacle avoidance

## Abstract

**Introduction:**

An age-related decrease in the ability to exploit the abundant degrees of freedom of the body, referred to as motor flexibility, leads to a heightened fall risk. The present study investigated motor flexibility to stabilize the toe position during obstacle crossing in older adults and its correlation with the magnitude of foot elevation.

**Methods:**

Twenty-six older adults (70.9 ± 7.4 years old) and 21 younger adults (25.4 ± 5.0 years old) walked and crossed an obstacle, during which the dominant limb was always the leading limb. An uncontrolled manifold (UCM) analysis was used to quantify the flexibility during obstacle crossing as the synergy index, with the vertical toe position being regarded as the performance variable and the segment angles of the lower limbs as the elemental variables.

**Results and discussion:**

The results showed that older participants had a significantly lower synergy index for the trailing limb before the moment of obstacle crossing than younger participants, suggesting reduced flexibility in part. The results also showed that, regardless of age, foot elevation was negatively correlated with the synergy index, suggesting that a so-called “conservative strategy” (i.e., a tendency to show extraordinarily high foot elevation to ensure collision avoidance) may be related to their reduced motor flexibility.

## Introduction

Precisely controlling the toe position is critical to avoiding tripping and destabilization when stepping over an obstacle (1–3). Experimental studies have produced contradictory findings about the foot elevation of older adults in the vertical dimension in stepping over an obstacle. In some studies, older adults showed a lower foot elevation (i.e., lower clearance height), which could lead to tripping (4, 5). In other studies, older adults exhibited a higher foot elevation while stepping over an obstacle (2, 6, 7). Although such behavior, which has been referred to as “a conservative strategy” (6, 7) is helpful to avoid tripping as a result of creating a greater safety margin, this behavior could also result in destabilization, which could also lead to falls (8–11). These findings suggest that not only much lower foot elevation but also much higher foot elevation during stepping over an obstacle of height could lead to falls due to tripping or destabilization, respectively.

Precisely controlling the foot elevation to step over an obstacle may be achieved with flexible control of body parts, termed *motor flexibility*. Motor flexibility is defined as “the ability to synergistically control the abundant degrees of freedom (DoFs) of the body to ensure stable performance of a task” (12). For several reasons, such as the existence of neuromotor noise, having different sets of joint coordination is beneficial for accomplishing a same task goal, rather than mastering coordination of a single set of joints (13). For example, when reaching for a target with the fingertips, a combination of shoulder, elbow, and wrist joint angles (i.e., the element variables) is controlled to stabilize the fingertip position [i.e., the performance variable (14)]. Even when an obstacle exists on the usual reaching trajectory, various movement patterns can be used to successfully reach for a target by altering the combination of these joint angles (14). When elevating the foot to a certain height, a combination of hip, knee, and ankle joint angles is controlled to stabilize the foot position. Even when one joint is fixed or cannot be used due to injury, various movement patterns can be used to succeed in elevating to the same height by altering the combination of these joint angles. Considering these cases, it is natural to consider that motor flexibility would be the key to achieving precise control of foot elevation for stepping over an obstacle.

Several studies have reported age-related decreases in motor flexibility. Verrel et al. (15) investigated the age-related difference in motor flexibility in a manual pointing task. Uncontrolled manifold (UCM) analysis is a technique used to quantify motor flexibility by exploiting the abundant DoFs (i.e., the elemental variable) to ensure the critical variable (i.e., the performance variable) as the synergy index (12, 14, 16, 17). The higher synergy index means greater motor flexibility (12, 14). Verrel et al. (15) showed that the synergy index related to coordination across joints to stabilize fingertip position in older adults was significantly lower than that in younger adults, despite the fact that the endpoint precision was similar between the two age groups. Consistently, Hsu et al. (18) also showed age-related decreases in motor flexibility for maintaining balance during upright standing. The results showed that the synergy index during maintaining balance in older adults was lower than that in younger adults. These results suggest that motor flexibility to stabilize the performance variable is decreased in older adults.

Recently, Yamagata et al. (11) reported an age-related decrease in motor flexibility to maintain stability during stepping over an obstacle and its relation to higher foot elevation. In Yamagata et al., healthy older adults walked and crossed an obstacle with a fixed height (8 cm). Yamagata et al. used UCM analysis, in which the center of mass (COM) position was set as the performance variable, while segment angles of the body were set as elemental variables, and they calculated the synergy index during stepping over an obstacle with both the leading and trailing limbs. The results showed that the synergy index was lower for the trailing limb than for the leading limb, suggesting less stability when an obstacle is stepped over with the trailing limb. Notably, they found that the synergy index was negatively correlated with the maximum foot elevation in the trailing limb. The findings of Yamagata et al. are particularly important for the present study because they indicate that age-related instability during stepping over an obstacle is associated with lower motor flexibility, as well as higher foot elevation, i.e., the conservative strategy.

The present study investigated motor flexibility for stabilizing the toe position during obstacle crossing in older adults and its correlation with the magnitude of clearance height. The present study was designed to extend the knowledge obtained by Yamagata et al. (11). There were two main differences between Yamagata et al. and the present study. First, whereas Yamagata et al. used the COM position as the performance variable to examine motor flexibility to maintain stability of the whole body, we used the toe position in the vertical direction to examine motor flexibility to achieve precise control of the toe height. Second, while Yamagata et al. examined motor flexibility only in older adults, the present study compared performances of older adults with those of younger adults to clearly show that the data obtained from the older participants represent age-related changes.

To investigate whether motor flexibility to ensure foot elevation is lower in older adults than in younger adults, we conducted the UCM analysis in which toe height was used as the performance variable. Based on the previous studies showing an age-related decrease in the adjustment of foot elevation with both the leading and trailing limbs (2, 6), we conducted UCM analysis for the leading and trailing limbs. Furthermore, UCM analysis was conducted not only at the moment of obstacle crossing but also for the whole swing phase. Although the toe position at the moment of obstacle crossing is critical, movement during the swing phase, especially before stepping over an obstacle, affects that position (2, 4, 8). Based on these studies, we conducted UCM analysis for the whole swing phase to examine whether there would be age-related decline in motor flexibility for controlling the toe height during the swing phase before stepping over an obstacle (i.e., the pre-crossing phase).

The excessive foot elevation was not only related to a loss of balance in older adults, but also may be related to reduced motor flexibility to ensure foot elevation. A relevant previous study showed that inactive older adults, who walked less frequently in their daily activities, showed more stereotyped walking patterns than active older adults (19). This implies that inactive older adults have less opportunity to adjust their movements in response to environmental changes, resulting in more stereotyped (less flexible) behavior. If this is the case, then we assume that adopting a conservative strategy allows older adults to perform a one-size-fits-all pattern (i.e., exaggerated movement pattern) and could lead to the decline in motor flexibility. Alternatively, independent of the age-related decline in physical/motor functions, highly elevating the foot with extreme joint angles (i.e., greater hip, knee, and ankle flexions) leads to reduced joint range of motion (ROM), resulting in lower motor flexibility. Therefore, we considered that a conservative strategy may be associated with the decline in motor flexibility.

There were two hypotheses in the present study. The first hypothesize was that the synergy index (ΔVz) is lower in older adults than in younger adults with both the leading and trailing limbs in the pre-crossing phase including at the moment of obstacle crossing. The second hypothesis was that, consistent with the findings of Yamagata et al. (11), higher foot elevation (i.e., a conservative strategy) is associated with less motor flexibility. Specifically, we expected that foot clearance (equivalent to foot elevation), calculated as the distance between the toe and the obstacle, was negatively correlated with the ΔVz with both the leading and trailing limbs.

## Methods

### Participants

Twenty-six older adults (12 males and 14 females, 70.9 ± 7.4 years) and 21 younger adults (16 males and 5 females, 25.4 ± 5.0 years) participated. The sample sizes were determined based on similar studies (11, 20, 21) and *a priori* power analysis assuming a correlation analysis. We calculated the sample size based on the power analyses with G^∗^Power: effect size = 0.5, significant threshold (*α*) = 0.05 and power levels (1-*β*) = 0.8. The effect size 0.5 was decided based on similar previous report (11). In the sensitivity power analysis for the *t*-test, the effect size (Cohen's d) was calculated to be 0.84: sample size = 47 (21 for younger adults and 26 for older adults) *α* = 0.05 and 1-*β*= 0.8. We checked on a self-reported basis that all participants had normal or corrected-to-normal vision, no current musculoskeletal injuries, and no neurological disorders. For older participants, cognitive and mobility function were assessed using the Mini-Mental State Examination [MMSE (22)] and the Timed Up and Go (TUG) test (23). No cognitive impairment (MMSE score ≧ 24 points (22); and mobility impairment [TUG score < 13.5 s (24)] were inclusion criteria for older adults. The study was approved by the Ethics Committee of Tokyo Metropolitan University, Japan (H3–129). All methods were carried out in accordance with relevant guidelines and regulations. Written informed consent was obtained from all participants in accordance with the Ethics Committee of Tokyo Metropolitan University and the Declaration of Helsinki.

### Procedures

**Figure 1:**
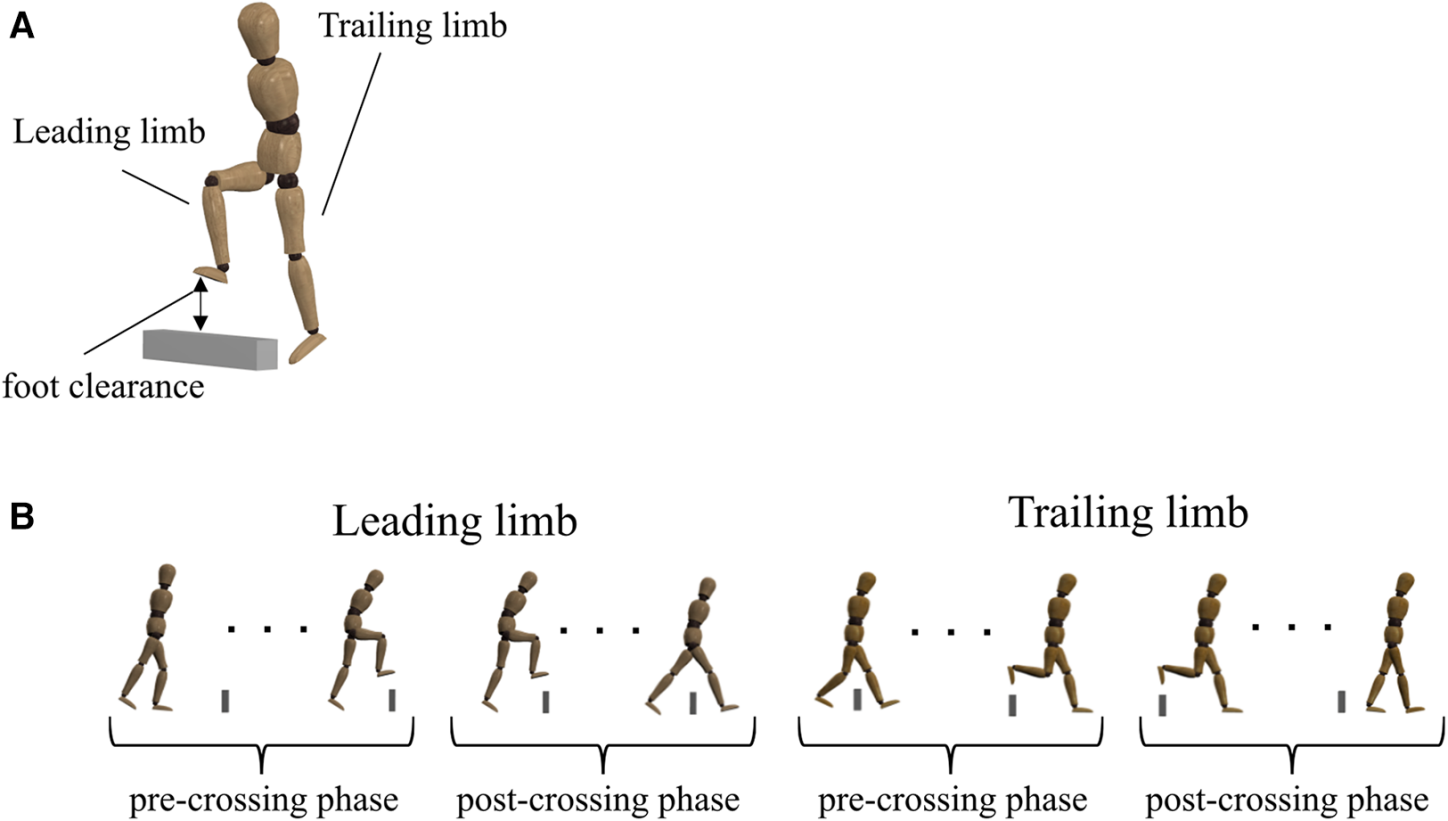
(**A**) participants walked and crossed an obstacle 20 times, during which the dominant limb was always the leading limb. Foot clearance was calculated as the vertical (**V**) distance between the markers on the obstacle and on the toe at the moment of obstacle crossing. Because of anthropometric differences between older and younger adults, each parameter was normalized to the participant's leg length to account for between-subject differences. (**B**) Definition of the pre-crossing and post-crossing phases for the leading and trailing limbs. The moment of obstacle crossing was defined as the moment when the toe marker crossed the obstacle marker in the anterior–posterior (AP) direction. Each phase was shown as the normalized movement time. In the pre-crossing phase, 0% represented toe-off, and 100% represented the moment of obstacle crossing. In the post-crossing phase, 0% represented the moment of obstacle crossing, and 100% represented heel contact.

The experiment was conducted in a 6.7 m × 4.9 m room at Tokyo Metropolitan University. The obstacle consisted of two aluminum poles (1.91 m tall and 0.03 m in diameter) and a horizontal wooden bar (1.2 m wide and 0.05 m in diameter) covered with a padding. A bubble wrap was used for padding to prevent injuries in collisions. The obstacle height was set at 8 cm including the padding. Participants walked along a straight 4 m path and crossed the obstacle—which was located 3 m from the starting position—at a comfortable pace, repeated 20 times. Sampling of 15–20 trials were recommended for reliable quantification of the UCM analysis in locomotor task (25). In fact, relevant studies using the UCM analysis collected the data with similar numbers of repetition (e.g., 20 trials in (11). A smaller number of repetitions is not ideal for the UCM analysis because the standard deviation of the variance components does not stabilize. In each trial, they were asked to cross the obstacle with their dominant limb (defined as the kicking foot) as the leading limb [(26); Figure 1A]. The first step of gait initiation was chosen by each participant. There were no any other instructions for crossing (e.g., to cross as low as possible). Trials in which participants accidentally contacted an obstacle were regarded as unsuccessful trials and were excluded from the analyses. An additional trial was run until the number of successful trials reached a total of 20. In total 940 trials, there was only a single trial in which a collision—by an older participant—occurred. Prior to performing the main trials, participants performed five practice trials to familiarize themselves with the task, and the starting position was adjusted so that the leading limb was the dominant limb.

### Data collection

Before the experiment, participants' heights and leg lengths were measured in cm, and their weights were measured in kg. A three-dimensional (3D) motion analysis system (OQUS 300, Qualisys, Sweden) with 17 cameras was used to analyze the kinematic data relating to the behavior of stepping over an obstacle. Twenty-six reflective markers were placed on both sides of the body and trunk as follows: ear canal, acromia, olecranon processes, styloid processes of the ulnae, 7th cervical, 10th thoracic vertebra, xiphoid process, manubrium sterni, anterior superior iliac spine, posterior superior iliac spine, greater trochanter, lateral femoral condyles, lateral malleolus, calcaneus, and second metatarsal. Two additional reflective markers also were placed on the top right and left edges of the obstacle. The sampling frequency was 120 Hz. The 3D data for all markers were low-pass filtered at 6 Hz with a fourth-order Butterworth algorithm. All data analysis was conducted using MATLAB (R2022a, MathWorks Inc., Natick, MA, USA).

We defined the moment of obstacle crossing as the moment when the marker on the toe crossed the marker on the obstacle in the anterior–posterior (AP) direction. All kinematic parameters, including UCM analyses, calculated the swing phase (from toe-off until heel contact) when the leading and trailing limbs crossed an obstacle (e.g., the leading limb and the trailing limb). Toe-off and heel contact events were determined using the displacements of the toe and heel markers in the vertical (V) direction, respectively (27). Specifically, toe-off occurs at the lowest position of the toe marker, while heel contact occurs at the lowest position of the heel marker. We divided the swing phase into two phases (e.g., pre-crossing and post-crossing phases) and normalized each phase to 100% (Figure 1B). In the pre-crossing phase, 0% represented toe-off, and 100% represented the moment of obstacle crossing. In the post-crossing phase, 0% represented the moment of obstacle crossing, and 100% represented heel contact. Because the moment of obstacle crossing is the most critical for avoiding stumbling, we divided the swing phase based on the moment of obstacle crossing.

### Parameters

We calculated parameters representing the movement of stepping over an obstacle for both the leading and trailing limbs: foot clearance (Figure 1A). Foot clearance was the vertical (V) distance between the markers on the obstacle and on the toe at the moment of obstacle crossing. The center of mass (COM) position of the whole body was calculated as the sum of body segmental COM based on the previous study (28). The COM position at the moment when the COM crossed the marker on the obstacle in the AP direction was used for the calculation of the gait speed. We also calculated the toe-displacement variability in the V direction during the pre-crossing phase and the post-crossing phase. First, toe displacements were calculated. The V toe displacements were expressed relative to the ground. Then, the toe-displacement variabilities were calculated by the standard deviations of toe displacements across 20 trials and averaged for every 20% of the movement time. Each parameter was normalized to the leg length to account for differences between subjects.

### UCM analysis

**Figure 2:**
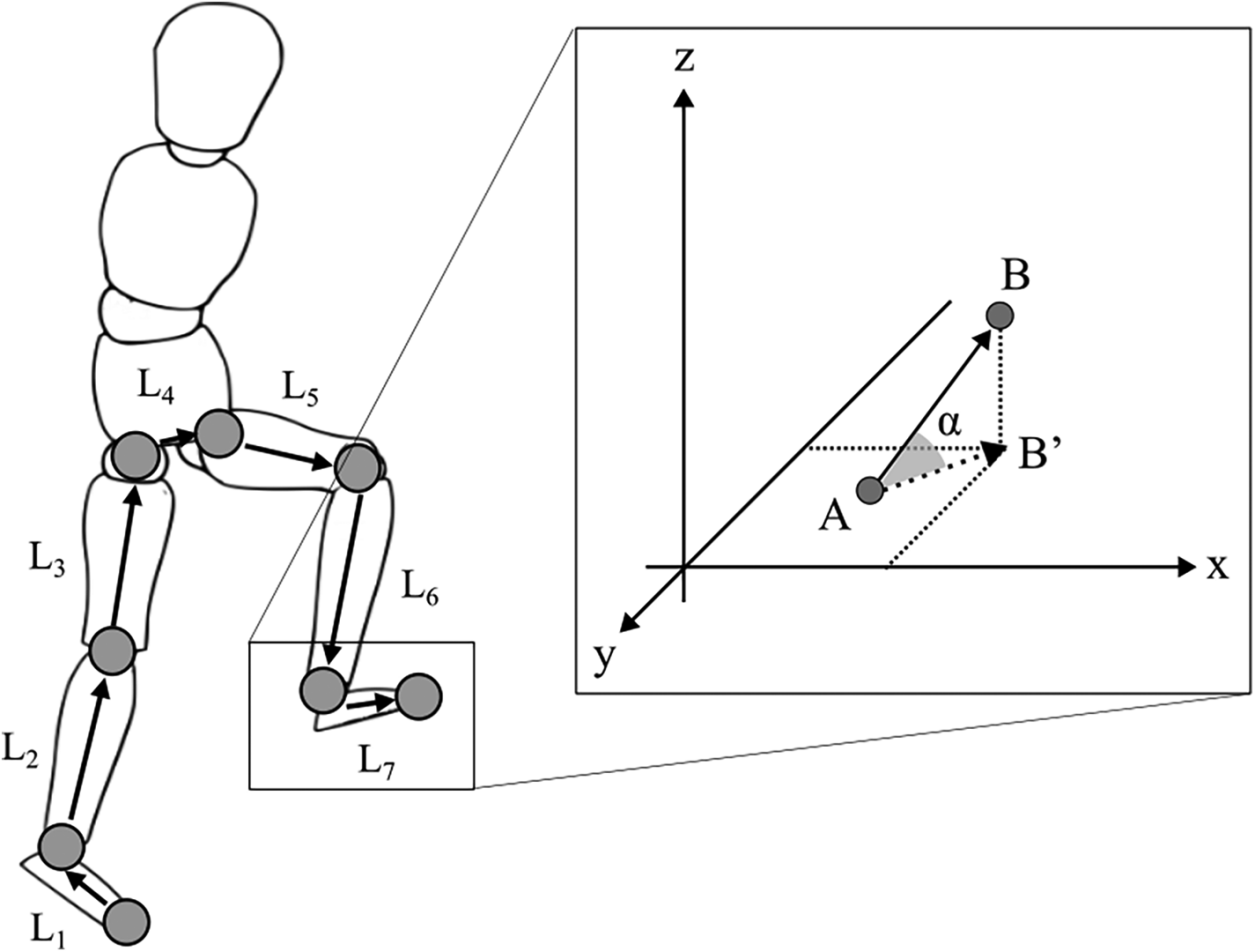
Geometric model for UCM analysis. L represents each segment length. The segments were defined by the following markers: each foot (L1, L7) is from the second metatarsal to the lateral malleolus; each shank (L2, L6) is from the lateral malleolus to the lateral femoral condyles; each thigh (L3, L5) is from the lateral femoral condyles to the anterior superior iliac spine; and the pelvis (L4) is connected to the right and left anterior superior iliac spine. The elevation (*α*) angles for each segment were calculated as shown in the figure on the right, with the starting point of each segment as A and the endpoint as B from the x-axis of the global reference frame.

The ability to exploit the abundant DoFs of the body when stepping over an obstacle was evaluated using UCM analysis (11, 14, 17, 29). For the UCM analysis, kinematic data were time-normalized (see *data collection*). We used toe position in the V direction as the performance variable because the motor flexibility to stabilize the toe height played an important role in avoiding collisions. The elemental variables for the performance variable were the elevation angle of each lower limb segment (Figure 2). We defined the seven body segments (right/left foot, right/left shank, right/left thigh, and pelvis). The elevation angles were calculated as the angles between each segment vector and the horizontal plane (Figure 2). The reason for using segment angles instead of joint angles was that many prior studies on walking deal with segment angles rather than joint angles (30–34). Then, we created a geometric model that has 7 DoFs for the V direction (TOE_V_) (30, 35), as follows:

$$\mathrm{TO}E_{V}\;=\;L_{1}\sin\alpha_{1}\;+\;L_{2}\sin\alpha_{2}\;+L_{3}\sin\alpha_{3}\;+\ldots \;+\;L_{7}\sin\alpha_{7}$$

where L is the length of each segment, and *α* is the elevation angle.

A Jacobian (*J*) matrix was calculated to obtain a linearization approximation of the geometric model of the performance variable. *J* is the matrix of partial derivatives of changes in the performance variable with respect to the elemental variables, and the null space (*E*) was calculated to provide basis vectors spanning the *J*. The *E* represented *n-d* vectors by the number of dimensions of the elemental variables (*n*) and the performance variable (*d*). In this study, *n* = 7 and *d* = 1 were used for the *TOE_V_*. At every point of the crossing phase, the differences between the elemental configurations (θ) and their mean ($\bar{\theta }$) were projected onto the null space:

$$\theta_{UCM}\;=\;\overset{n-d}{\underset{i}{\sum }}\;(\theta -\;\bar{\theta })\;\cdot \;E_{i},$$

and onto a component orthogonal to this subspace:

$$\theta_{ORT}\;=\;(\theta -\;\bar{\theta })\;-\;\theta_{UCM.}$$

V_UCM_ and V_ORT_ were calculated as the variance of the *θ*_UCM_ and *θ*_ORT_ and normalized by DoFs within the UCM subspace and ORT subspace, respectively. These variances were calculated as the average of the trial:

$$V_{UCM}\;=\;\frac{1}{(n\;-d)\;\;\cdot \;N}\;\sum (\theta_{UCM}{)}^{2},$$

$$V_{ORT}\;=\;\frac{1}{d\;\cdot N}\sum (\theta_{ORT}{)}^{2},$$

where *N* was the trial number. The V_UCM_ is a variance that has no effects on the performance variable, and the V_ORT_ is another variance that negatively affects the performance variable. We also calculated the synergy index (ΔV) as the proportion of the difference between V_UCM_ and V_ORT_:

$$\Delta V\;=\;\frac{V_{UCM}\;-\;V_{ORT}}{V_{UCM}\;+\;V_{ORT}}.$$

A higher ΔV reflects more solutions (i.e., one's goal by combining DoFs in different ways) utilized to stabilize the performance variable. For statistical analysis, ΔV was transformed using Fisher's z-transformation (ΔVz) (36).

We used three outcomes (V_UCM_, V_ORT_, and ΔVz) to investigate differences in the motor flexibility of older and younger adults. All data obtained from the UCM analyses computed at each point in normalized time were then averaged for every 20% of the normalized time (20, 21).

### Statistical analysis

Before statistical analyses, the data were tested for statistical assumptions of normality. The height, weight, leg length, gender ratio, normalized foot clearance, normalized toe displacement variabilities, V_UCM_, and V_ORT_ did not achieve normality for participants. For V_UCM_ and V_ORT_, we used log-transformation (34). A Mann–Whitney *U*-test was used to compare participants' characteristics (height, weight, and leg length), excluding gender. A Pearson's chi-squared test was conducted to compare the gender ratio. A Mann–Whitney *U*-test was also performed on the normalized toe clearance and normalized toe-displacement variabilities. Student's *t*-test was performed on the gait speed, the ΔVz, Log(V_UCM_), and Log(V_ORT_) to compare older and younger adults. We showed the results of the Log(V_UCM_) and the Log(V_ORT_) in the Supplementary Figure S1, not in the main manuscript, considering the preliminary analyses showing that these parameters represented no trend for discussion with our main results. A Spearman's correlation analysis was used to examine the relationship between the ΔVz in the V direction for all participants and the normalized foot clearance under the leading and trailing limbs. Statistical analyses were performed using IBM Statistical Package for the Social Sciences (SPSS) for Windows, version 28 (IBM Corp., Armonk, N.Y., USA). Statistical significance was set at *p* < 0.05.

## Results

### Participant details

Participants' characteristics are summarized in Table 1. The gender ratio was significantly different between older and younger adults. Height was significantly lower in older adults than in younger adults. No significant difference between older and younger adults was found in weight or leg length. All older adults were right-limb dominant, while 19 of 21 younger adults were right-limb dominant. Gait speed was significantly lower in older adults than in younger adults.

**Table 1:** Participants’ details: mean ± standard deviation.

	Younger adults (n = 21)	Older adults (n = 26)	*p-*value
Gender (male/female)^a^	16/5	12/14	*p* = .037
Age (years)^b^	23.28 ± 4.95	70.90 ± 7.43	*–*
Dominant limb (right/left)	19/2	26/0	*–*
Height (cm)^b^	168.97 ± 7.58	160.82 ± 9.54	*p* = .009
Weight (kg)^b^	60.57 ± 10.46	57.67 ± 11.64	*p* = .600
Leg length (cm)^b^	76.04 ± 3.89	74.76 ± 4.67	*p* = .472
Gait speed (m/s)^c^	1.28 ± 0.17	1.11 ± 0.15	*p* < .001
TUG (s)	*–*	7.27 ± 1.21	*–*
MMSE (points)	*–*	29.43 ± 1.21	*–*

TUG, timed up and go test; MMSE, mini-mental state examination.

^a^ Pearson's chi-square test.

^b^ Mann–Whitney *U*-test.

^c^ Student's *t*-test.

### Lower limb movement when stepping over an obstacle

**Figure 3:**
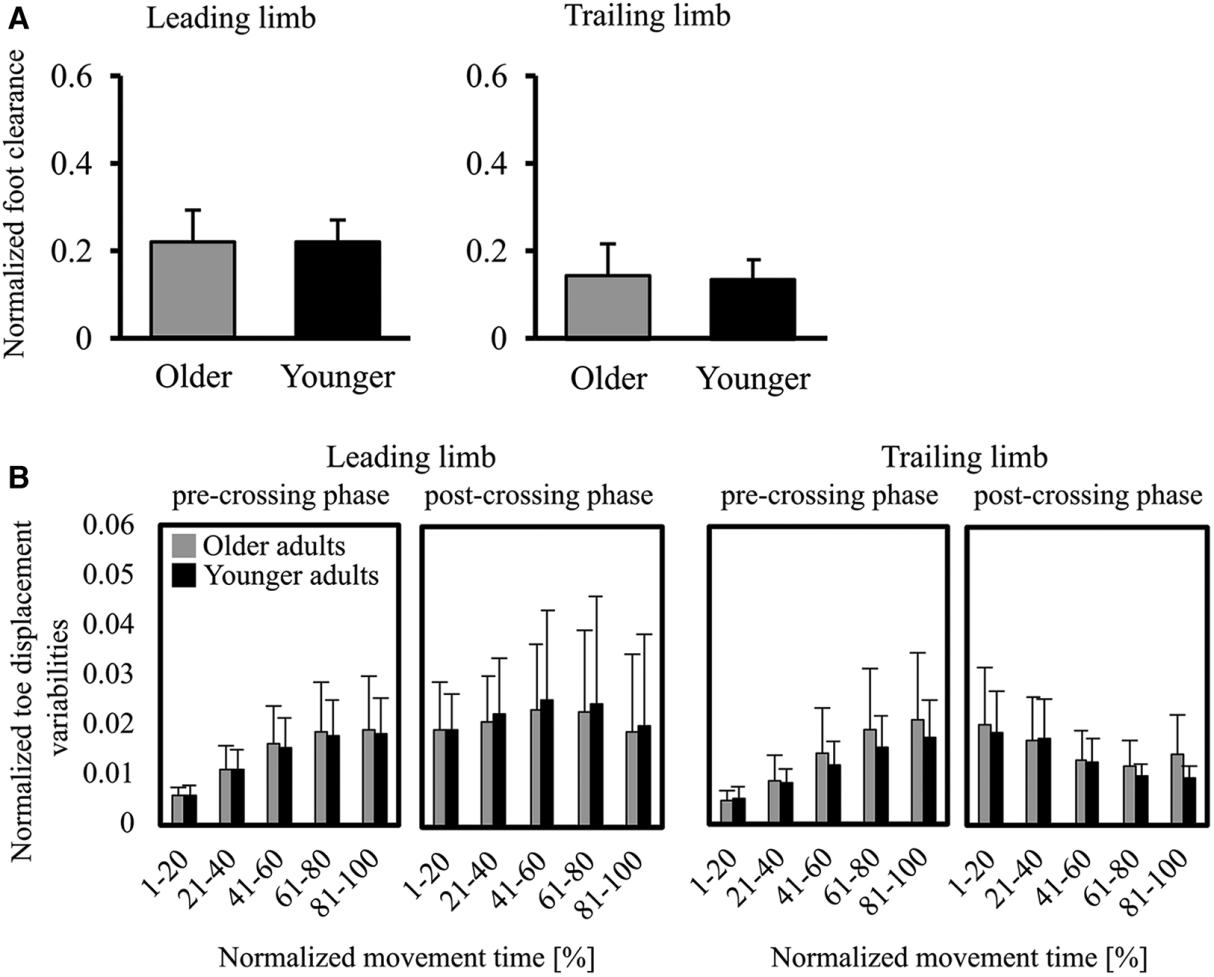
(**A**) graphs of the normalized foot clearance (foot clearance/leg length) in the leading/trailing limbs. Error bars represent the standard deviation. (**B**) Graphs of foot displacement variabilities for the V direction. The toe-displacement variabilities were calculated by the standard deviations of the toe displacement across 20 trials and then averaged for every 20% of the movement time. Error bars represent the standard deviation among participants.

Figure 3A shows parameters representing the movement of stepping over an obstacle—the normalized foot clearance in the leading and trailing limbs. No significant differences between older and younger adults were found in the leading (*p* = .653) and trailing (*p* = .669) limbs. Furthermore, the normalized foot clearance for each trial at each participant are shown in Supplementary Figures S2, S3. Visual inspection of the figure shows that no trend for foot clearance in the leading and trailing limbs to change from the first half of the trial to the second half was observed in older and younger adults. Figure 3B shows the toe displacement variabilities in the V direction. There were no significant differences between older and younger adults in the leading and trailing limbs during all phases.

### The synergy index by UCM analysis

**Figure 4:**
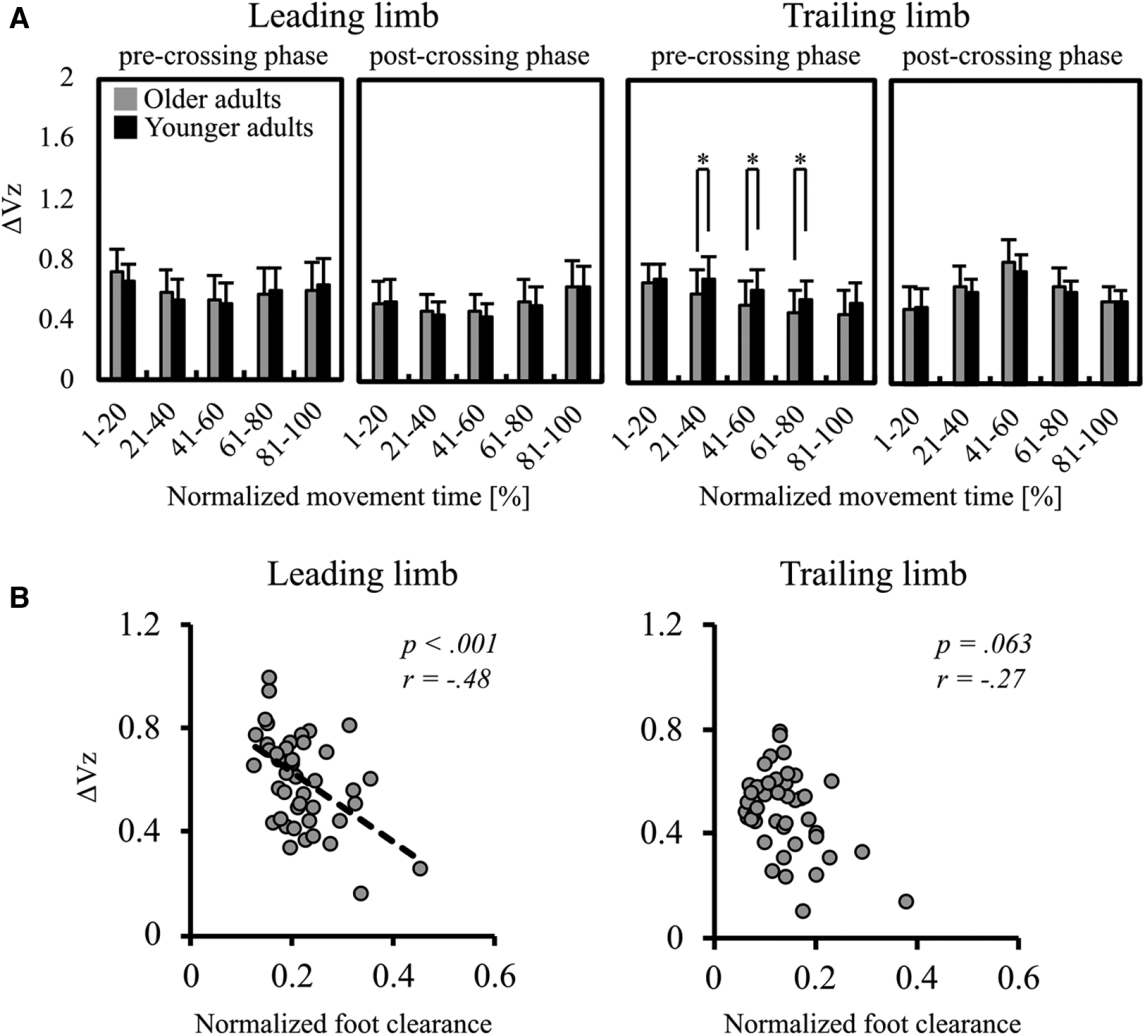
(**A**) mean ΔVz in the leading and trailing limbs. The two left panels indicate the ΔVz in the leading limb, and the two right panels indicate the ΔVz in the trailing limb. Error bars also represent the standard deviation among participants, and * indicates a significant difference. (**B**) The results of Spearman's correlation analysis for examining the relationship between the ΔVz for all participants and the normalized foot clearance of the leading (left panel) and the trailing (right panel) limbs.

The mean ΔV_Z_ is shown in Figure 4A. In the trailing limb during the pre-crossing phase, the ΔV_Z_ in older adults was significantly smaller than that in younger adults at 21%–40% [*t*(45) = 2.03, *p* = .048], 41%–60% [*t*(45) = 2.41, *p* = .020], and 61%–80% [*t*(45) = 2.20, *p* = .034]. No significant difference was found in the trailing limb during the pre-crossing phase at 1%–20% and 81%–100% and during the post-crossing phase at all normalized movement times. No significant difference was found in the leading limb during all phases.

Figure 4B shows the results of correlation analysis between the ΔV_Z_ at the moment when stepping over the obstacle and foot clearance. Foot clearance was negatively correlated with the ΔVz in the leading limb (*r* = −.48, *p* < .001). However, no significant correlation was found in the trailing limb (*r* = −.27, *p* = .063).

## Discussion

The results showed that, for the trailing limb, during the pre-crossing phase, the ΔV_Z_ was significantly lower in older adults than in younger adults (Figure 4A). This partly supports the first hypothesis, in that, at least for specific limb in specific phases, motor flexibility was decreased in older adults. The results also showed that, regardless of age, foot clearance was negatively correlated with the ΔVz in the leading limb (Figure 4B). This supports the second hypothesis, that foot clearance is negatively correlated with the ΔVz.

UCM analysis showed that older participants had a reduced ΔVz with the trailing limb in the 21%–80% pre-crossing phase. Yamagata et al. (11) selected the COM position as the performance variable for the UCM analysis. In the present study, toe height was selected as the performance variable. Our findings provide insight into motor flexibility, particularly from the perspective of precisely controlling toe height. It is possible that, independent of the age-related decline in physical/motor functions, highly elevating the foot with extreme joint angles (i.e., greater hip, knee, and ankle flexions) leads to reduced ROMs, resulting in lower motor flexibility. However, our results showed that normalized foot clearance was not significantly different between older and younger participants (Figure 3A), implying that the reduced motor flexibility in the present study was not a by-product of extreme joint angles with reduced joint ROMs. Instead, we assumed that an age-related decline in motor flexibility would be involved. Moreover, the age-related decrease in motor flexibility for stabilizing toe height was also limited before the moment of obstacle crossing (the pre-crossing phase). This is consistent with the previous study which used the reaching task (15). These results suggest that older adults were unable to adjust their flexibility just before the obstacle-crossing rather than at the most critical point (e.g., the moment of obstacle-crossing).

An age-related decrease in motor flexibility for stabilizing the toe height to step over an obstacle was observed only with the trailing limb. This was inconsistent with our hypothesis of a significantly lower ΔVz with both the leading and trailing limbs. A possible explanation for the result is that control of the leading limb was less susceptible to aging due to the availability of vision for its control. Motor flexibility for stabilizing the COM position during walking was higher when vision was available than when vision was unavailable (37). Online visual information, particularly below the peripheral vision, is available for stepping over an obstacle with the leading limb, but not with the trailing limb. The effects of aging may be more pronounced when vision is less involved in the control of movement.

The decrease in the ΔV_Z_ in older adults would be due to a reduction in the movement patterns of combinations of joint angles. The ΔV_Z_ was decreased by a reduction in motor solutions or an increase in the variability of the performance variable—which reflects instability (14, 38). In the present study, the results of the toe-displacement variability (Figure 3B) showed that the accuracy of the toe height (the performance variable) did not differ between older and younger adults. Therefore, the present findings suggest that older adults ensured the accuracy of the performance variable across trials but utilized fewer motor solutions to stabilize the performance variable when stepping over an obstacle.

Correlation analysis showed that higher foot clearance was associated with less flexibility to stabilize the toe height in the leading limb. A conservative strategy would be observed frequently in older adults (2, 6–8) because the strategy is beneficial to minimize the risk of tripping. However, previous studies have indicated that the conservative strategy may disturb balance maintenance (8, 11). Using toe height as the performance variable in UCM analysis, the present results showed another disadvantage of taking a conservative strategy: higher foot elevation was associated with less ability to flexibly adjust toe height.

The results of correlation analysis may be interpreted bidirectionally. On one hand, a conservative strategy might prevent a person from fine-tuning his behavior in response to environmental changes, as a conservative strategy enables the avoidance of any characteristic of obstacles with a stereotyped behavior. Therefore, a repeated conservative strategy may induce less flexibility. On the other hand, age-related decrease in motor flexibility may lead older adults to adopt a conservative strategy. Tripping with an obstacle has a major impact on maintaining stability, particularly for those who are less capable of recovering trip-induced destabilization (39). They would have no choice but to take a conservative strategy (higher foot elevation to avoid collision with an obstacle). Future studies are necessary to address more clearly what the association between higher foot clearance and lower motor flexibility for stabilizing the toe height means.

For successful obstacle crossing, not only the toe height but also the other critical variables need to be controlled. Incorrect foot placement before stepping over an obstacle (i.e., too far or too close to it) could lead to tripping (4, 6, 40, 41). Moreover, obstacle crossing involves not only successful foot elevation but also maintaining stability. In our study, we focused only the V direction of the toe position to address the present question. Future studies which consider other critical variables to achieve successful obstacle crossing are necessary to folly explain the how relevant body parts are to be coordinated.

The characteristics of the movement during stepping over an obstacle showed a few insights. First, the mean normalized foot clearance indicated similarities between the previous and present studies (2, 7). In the present study, because the obstacle used in this study was of low height (8 cm), no difference between older and younger adults was found in normalized foot clearance. Previous studies have showed no difference in the foot clearance of older and younger adults on obstacles of low height; however, as the height of the obstacle increased, older adults had greater foot clearance than younger adults (2, 7). Second, the results of the normalized foot clearance for each trial at each participant showed that no learning effects or adaptation in toe clearance over the repetitions of 20 trials both the leading and trailing limbs. We therefore believe this has no impact on the results of the UCM analysis. Third, the gait speed results showed slower speed for older participants than younger participants. This indicated that older participants had low gait ability compared to younger adults. However, we believe this does not have a significant impact on the results, based on a previous study demonstrating that the gait speed does not affect the results of the UCM analysis (42).

This study had two limitations. First, the height of the obstacle was fixed at 8 cm. Repetition in stepping over an obstacle of the same height was necessary to conduct the UCM analysis. Although the UCM analysis allows researchers to quantify the coordination of elemental variables toward a particular goal, it is only applicable to the multiple measurements of the same movement. It cannot be applied to movements on stepping over different heights of obstacles. However, the use of a constant height for all participants means that the impact of the obstacle height could be different based on participants’ leg lengths. The significantly lower height of the older participants than younger participants means that the task could be of relatively greater difficulty for older adults. We believe that the impact would be relatively small, if any, given that the obstacle height of 8 cm is low enough. Second, in the present study, the older adults had high mobility function. Table 1 shows that all older participants were above the TUG cutoff value. However, the age-related decrease in motor flexibility should have impacted more severely those with decreased physical function (12, 43). In fact, Yamagata and colleagues reported that older adults with histories of falling showed less kinematic synergy during walking (43).

In conclusion, our results showed that motor flexibility for stabilizing the toe height of older adults was lower than that of younger adults, at least with the specific limb in pre-crossing phases. These results suggest that older adults may have less flexibility in adjusting to environmental changes and unexpected perturbations (18, 44, 45, 45) when stepping over an obstacle. In addition, regardless of age, a conservative strategy was associated with decreased motor flexibility in the leading limb. A conservative strategy may not necessarily be useful, in that it may induce less flexibility in response to environmental changes.

## Data Availability

The raw data supporting the conclusions of this article will be made available by the authors, without undue reservation.
