# Supplementary material for: Motor flexibility to stabilize the toe position during obstacle crossing in older adults: an investigation using an uncontrolled manifold analysis
Source: Front Sports Act Living. 2024 Mar 22;6:1382194. doi: 10.3389/fspor.2024.1382194 (PMC10995316; doi:10.3389/fspor.2024.1382194)
Supplement: Supplementary file 1 [file Datasheet1.pdf]

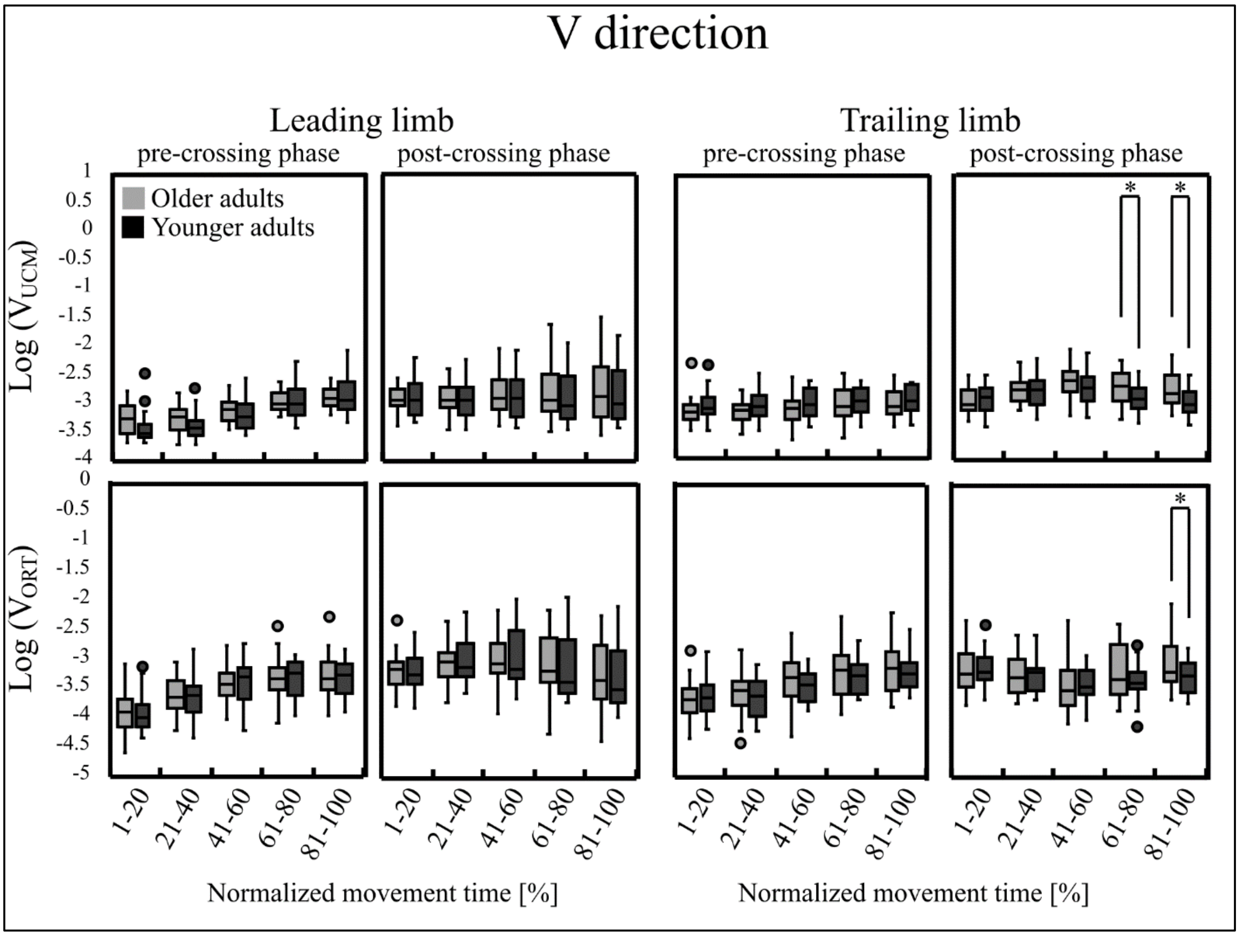

Supplementary Figure S1. Graphs of the mean Log(V_{UCM}) and Log(V_{ORT}) in the V direction. The top four panels indicate the Log(V_{UCM}), and the bottom four panels indicate the Log(V_{ORT}). Error bars represent the standard deviation among participants. * indicates a significant difference. In the trailing limb during the post-crossing phase, the Log(V_{UCM}) in older adults was significantly higher than that in younger adults at 61–80% ($t(45) = -2.83$, $p = .007$) and 81–100% ($t(45) = -2.66$, $p = .011$). In the trailing limb during the post-crossing phase, the Log(V_{ORT}) in older adults was significantly higher than that in younger adults at 81–100% ($t(45) = -2.23$, $p = .031$). No other difference between older and younger adults was found.
