# Supplementary material for: Motor flexibility to stabilize the toe position during obstacle crossing in older adults: an investigation using an uncontrolled manifold analysis
Source: Front Sports Act Living. 2024 Mar 22;6:1382194. doi: 10.3389/fspor.2024.1382194 (PMC10995316; doi:10.3389/fspor.2024.1382194)
Supplement: Supplementary file 2 [file Datasheet2.pdf]

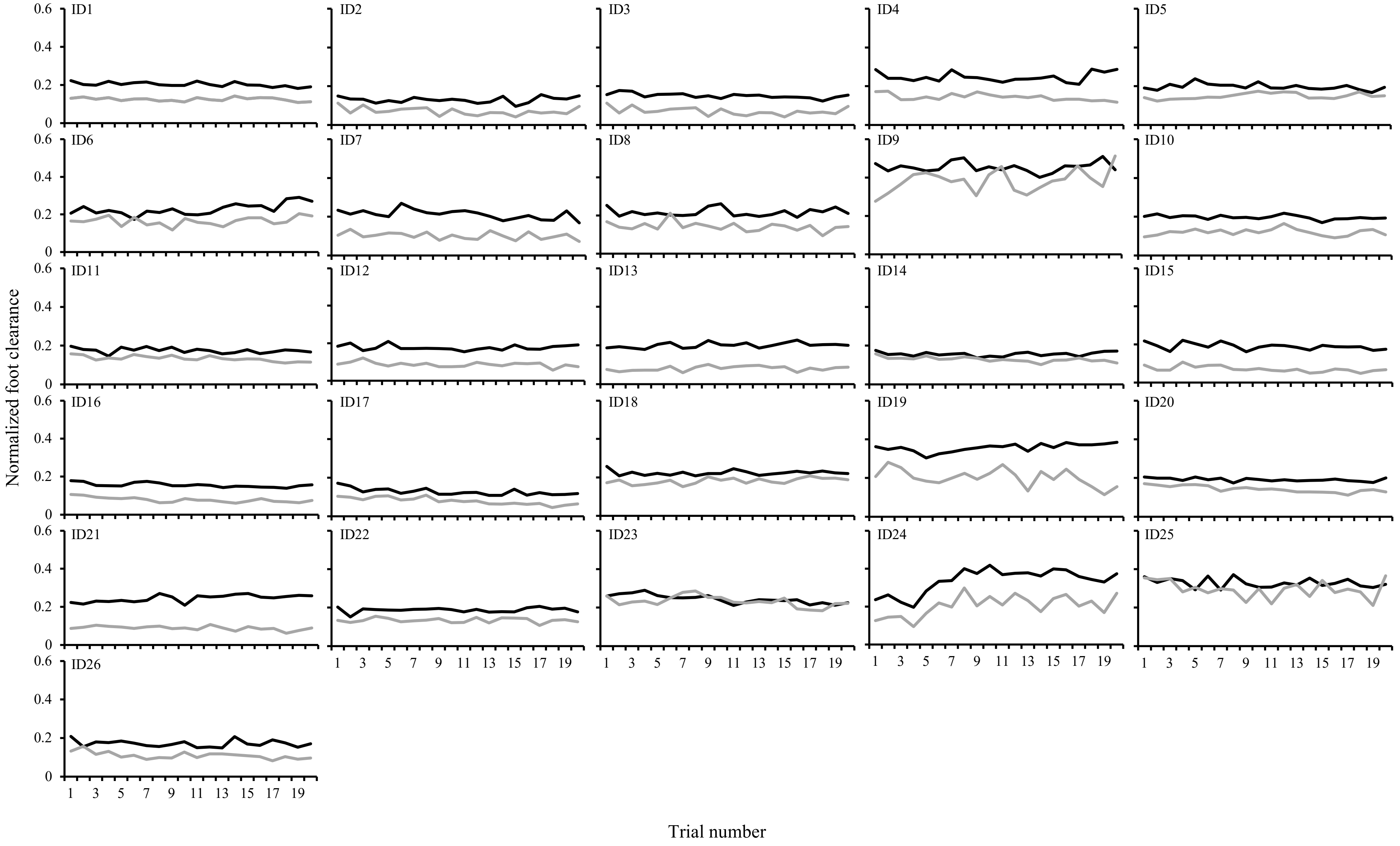

Supplementary Figure S2. Graphs of the normalized foot clearance for older adults. Black lines represent the leading limb. Gray lines represent the trailing limb.
